# Study of the Kinetics and Equilibrium of the Adsorption of Oils onto Hydrophobic Jute Fiber Modified via the Sol-Gel Method

**DOI:** 10.3390/ijerph15050969

**Published:** 2018-05-12

**Authors:** Na Lv, Xiaoli Wang, Shitao Peng, Huaqin Zhang, Lei Luo

**Affiliations:** 1 School of Environmental Science & Safety Engineering, Tianjin University of Technology, Tianjin 300384, China; tjutlvna@163.com (N.L.); pengshitaotj@163.com (S.P.); luoleitougao@sina.com (L.L.); 2 Laboratory of Environmental protection in Water Transport Engineering, Tianjin Research Institute for Water Transport Engineering, Ministry of Transport, Tianjin 300456, China; zhanghq@tiwte.ac.cn

**Keywords:** jute fiber, oil spill, the sol-gel method, hydrophobic, adsorption kinetics

## Abstract

A new kind of hydrophobic and oil sorbent based on jute fiber was successfully prepared by the integration of silica onto a fiber surface via the sol-gel method and subsequent hydrophobic modification with octadecyltrichlorosilane (OTS). Compared with the hydrophilic raw fiber, the modified fiber had a water contact angle (CA) of 136.2°, suggesting that the material has good hydrophobicity. Furthermore, the ability of oil in the oil/water system (taking diesel for example) to absorb was revealed by the kinetics, the isotherm equation, and the thermodynamic parameters. Adsorption behavior was kinetically investigated using pseudo first-order and pseudo second-order models. The data mostly correlated with the pseudo first-order model. The equilibrium adsorption at 298 K was assessed by using the Langmuir and Freundlich isotherm models. The Freundlich model had greater consistency with the experimental data. The obtained thermodynamic parameters demonstrate that the adsorption of diesel is spontaneous, favorable, and exothermic.

## 1. Introduction

Oil spills are one of the most seriously threats to the oceans. The commonly used methods for recovery of oil in such spills are the oil containment boom and linoleum methods. However, these oil recovery methods leave a thin layer of oil floating on the surface of the sea. Processes occurring with the disappearance of this layer such as drifting, diffusion, evaporation, emulsification, and sedimentation [1], severely contaminate the ocean and the surrounding environment.

Because of its low cost and high efficiency, an adsorption method is an effective way of removing oil from the ocean surface. A number of oil-absorbing materials for this method, such as natural inorganic materials [2], natural organic materials [3], and composite materials [4], have been applied to clean up oil spills. Natural organic materials such as wood chips [5,6], sugarcane bagasse [7] cotton [8,9], and jute fiber have been used because of their biodegradability. Jute fiber, a lignocellulosic natural fiber mainly composed of cellulose, hemicellulose (82%–85%), and lignin [10,11], has been developed as an adsorbent material by a number of researchers because of its low cost, durability, and eco-friendly properties. Many studies have been carried out to determine the adsorption performance of jute. For example, Gao et al. used jute modified with pyromellitic dianhydride to adsorb aniline in wastewater and obtained significant results [12]. Moreover, Arfaoui et al. attached ZnO nanorods to jute surfaces and then used fatty acids with a contact angle (CA) as large as 148° to conduct superhydrophobic treatment [13]. Most recently, Dong et al. inserted laccase into dodecyl gallate (CA of 111.5°) to modify a jute/polypropylene composite. The rupture strength increased with the assistance of a joint laccase branch; it thus met the requirements of compatibility with hydrophobic resins for producing high-performance fiber composites [14]. All of these studies show that modified jute fiber is an effective absorbing material and that it has potential use in oil-spill cleaning. However, little information is available in terms of the applications of modified jute fiber in ocean oil adsorption.

The main aim of this paper is to propose a preparation method for an environment-friendly oil sorbent that can be practically applied in the removal and recovery of oil spills on water surfaces. The objectives of this study were as follows: (1) to prepare and characterize modified jute fiber, including changes in surface structure and functional groups; (2) to measure the adsorption capacities of raw and modified fiber in pure water and oil systems; and (3) to interpret the kinetics, isotherms, and thermodynamics of diesel adsorption on the modified fiber.

## 2. Materials and Methods

### 2.1. Materials

Jute fiber from Hunan, China, was used as a main raw material. Octadecyltrichlorosilane (OTS, analytical grade) was purchased from Beijing McLean Reagent Ltd., China. Ethyl orthosilicate (TEOS, analytical grade) was obtained from Tianjin Xingfu Fine Chemical Research Institute, China. Glacial acetic acid, sodium hydroxide, anhydrous ethanol, ammonia, and hydrochloric acid (analytical grade) were supplied by Tianjin Wind Boat Chemical Reagent Technology Co. Ltd., Tianjin, China. Crude oil was from the Tianjin Water Transport Engineering Research Institute. Lubricating oil and peanut oil were purchased from a local market in Tianjin, China. The oil used for the experiment was a light diesel oil with a kinetic viscosity of 1.8 m^2^/s and a density of 0.82 g/cm^3^. It contains paraffins, naphthenes, and aromatic hydrocarbons of 9–18 carbon atoms.

### 2.2. Modification Method

#### 2.2.1. Pretreatment of the Jute Fiber

The raw materials were pulverized by a grinder, and then 20 to 40 mesh fiber particles separated using standard sieves of different meshes. The particles were then immersed in 7% NaOH solution over 4 h, and then dried at 50 °C in air for 6 h.

#### 2.2.2. Preparation of Surface SiO_2_ Particles under the Sol-Gel Method

TEOS, anhydrous ethanol, and deionized water were mixed to a ratio of 1:4:10, and 0.1 mol/L dilute hydrochloric acid solution was added to adjust the pH to 3–4. The pretreated fiber was immersed into the mixture and then stirred in a machine for 1 h to make it uniform. A 0.05 mol/L ammonia solution in water was used to make the mixture neutral, and stirring was continued for 2 h with shaking for 12 h. After it was shaken, it was allowed to stand for 4 h and then the fiber was dried. After this step, a layer of SiO_2_ particles was found attached to the surface of the jute fiber.

#### 2.2.3. Hydrophobic Modification

First, 1 mL of OTS was dissolved in 50 mL of n-hexane solution. The altered fiber was then immersed in the mixture for 2 h. Then, the fiber was taken out from the solution and washed with ethanol three times to remove the residual solution, and dried at 60 °C.

### 2.3. Characterizations

Fourier transform infrared (FTIR) spectra were recorded on a Tensor 37 FTIR spectrometer, using KBr pellets. Energy-dispersive spectrometry (EDS, Hitachi, Ltd., Tokyo, Japan) was then performed. Micrographs of the samples were obtained by scanning electron microscopy (SEM) using a Hitachi SU3500 (Hitachi, Ltd., Tokyo, Japan). Before SEM observation, the surface of the jute fiber was sputtered with gold by vacuum scanning. Water CA measurements were carried out using a DSA 100 (Krüss Company, Ltd., Hamburg, Germany), and the fiber was pressed into a sheet using a tablet press before measurement. Brunauer, Emmett and Telle (BET) Surface Area was measured by VacPrep 061 (Micromeritics, TrisStra II 3020, Atlanta, GA, USA) at 77 K.

### 2.4. Adsorbability Measurement

In this step, both of the adsorption properties of raw and modified jute fibers in pure water and the oil system were measured. According to ASTM F-726-12 [15], the adsorption capacity formula is expressed as follows:

(1) $$S_{w}=\frac{S_{wt}-S_{o}}{S_{o}}$$

where *S_w_* is the sorption rate (g (liquid)/g (sorbent)), *S_0_* is the quality of the jute fiber before sorption, and *S_wt_* is the quality of the jute fiber after sorption. We took 1 g of raw and modified jute fibers, immersed them in a beaker, and took measurements every 5 min. According to ASTMF-726-12, the test measures the rapid adsorption capacity (15 min soaking) and 24 h adsorption capacity. The water used for this test had a salinity of 35 ‰, which is the same as that of seawater.

### 2.5. Batch Experiments

A balanced oil–water mixture was pipetted into a separatory funnel, 15 mL of petroleum ether and 1 mL of 1 + 1 sulfuric acid (1mL sulfuric and 1mL water) were added, and then the funnel was shaken for 5 min. After the mixture was allowed to stand for 10 min, the lower aqueous phase was discharged. The organic phase was poured into a beaker, 1.2 g of anhydrous sodium sulfate was added, and then the mixture drained into a glass funnel using a glass rod. Afterward, the solution was filtered into a colorimetric tube, and 25 mL of petroleum ether added (the same process was repeated with the same volume of petroleum ether). Finally, the adsorbent was filtered, and the residual concentration of diesel analyzed by UV-Vis spectroscopy (UV-9600, Beijing General Analysis Instrument Co., Ltd., Beijing, China) at 510 nm.

Kinetic studies on adsorption were carried out by taking 50 mg of jute fiber in the oil/water solution at room temperature. Samples were withdrawn at different times (1–90 min), and the concentration of diesel measured.

At 293 K, the adsorption isotherm was obtained with the same procedure. The initial diesel concentrations were 5, 10, 15, 20, 25, and 30 g/L.

Adsorption thermodynamics were studied through batch experiments at different temperatures (293, 303, 313, and 323 K) using the same procedure.

The diesel adsorption capacity at equilibrium (Q) is calculated by the following formula:

(2) $$Q=\frac{(C_{0}-C_{e})V}{S}$$

where *C*_0_ and *C*_e_ are, respectively, the initial and equilibrium concentrations of diesel (g/L) at any time *t*. *V* is the volume of the solution (L), and *S* is the mass of the adsorbent (g).

### 2.6. Adsorption Kinetics

#### 2.6.1. Pseudo First-Order Model

The pseudo-first-order model is represented by the following equation [16]:

(3) $$\frac{dQ_{t}}{dt}=k_{1}\left(Q_{e}-Q_{t}\right)$$

When boundary conditions are reached, t=0,Q=0 and $t=t,Q=Q_{t}$, the equation can change to:

$$\ln(Q_{e}-Q_{t})=\ln Q_{e}-k_{1}t$$

which gives

(4) $$Q_{t}=Q_{e}-Q_{e}e^{-k_{1}t}$$

where *k*_1_ is the pseudo first-order rate constant; *Q_e_* and *Q_t_* are the adsorption capacities of the adsorbent at equilibrium.

#### 2.6.2. Pseudo Second-Order Model

The pseudo second-order model is represented as follows [17]:

(5) $$\frac{dQ_{t}}{dt}=k_{2}{\left(Q_{e}-Q_{t}\right)}^{2}$$

The linearized-integrated form of the equation is:

(6) $$Q_{t}=\frac{k_{2}Q_{e}^{2}t}{1+k_{2}Q_{e}t}$$

where *k*_2_ is the pseudo second-order rate constant.

#### 2.6.3. Intraparticle Diffusion Model

The intraparticle diffusion model can be used to analyze the removal of pollutants by an absorbent during a diffusion process. This is expressed as the following equation [18]:

(7) $$Q_{t}=k_{p}t^{0.5}+C$$

where *k_p_* is the intraparticle diffusion rate constant; and *C* is a constant related to the bounding layer thickness.

### 2.7. Adsorption Isotherm

#### 2.7.1. Langmuir Isotherm Model

The Langmuir isotherm model assumes that adsorption occurs at a specific uniform location on the adsorbent surface. According to this model, the adsorbent forms a molecular monolayer. The equation is as follows [19]:

(8) $$Q_{e}=\frac{K_{1}Q_{0}C_{e}}{1+K_{1}C_{e}}$$

where *Q*_0_ is the maximum adsorption capacity of the adsorbent (g/g); and *K_1_* is the Langmuir constant of equilibrium adsorption.

#### 2.7.2. Freundlich Isotherm Model

The Freundlich isotherm model assumes that multilayer adsorption takes place at heterogeneous surfaces with different adsorption energies and characteristics. Here, the adsorption of the surface is calculated by the following equation [20]:

(9) $$Q_{e}=K_{2}C_{e}^{1/n}$$

where *K_2_* (mg/g)(L/mg)^1/n^ is the Freundlich constant; and *n* is the adsorption intensity.

### 2.8. Adsorption Thermodynamics

The adsorption thermodynamics of the diesel adsorption process need to be further investigated. Various thermodynamic parameters such as enthalpy (Δ*H*), entropy (Δ*S*), and Gibbs free energy (Δ*G*) can be obtained by isothermal adsorption studies [21,22]. Δ*G* of adsorption can be represented by the classical van’t Hoff equation:

(10) $$\Delta G=-RTInK_{0}$$

where *K*_0_ can be calculated by the following equation:

(11) $$K_{0}=Q_{e}/C_{e}$$

The apparent enthalpy (Δ*H*) of adsorption and the entropy (Δ*S*) are calculated as follows:

(12) $$\ln(\frac{Q_{e}}{C_{e}})=\frac{\Delta S}{R}-\frac{\Delta H}{RT}$$

where Δ*G* is in (kJ/mol); Δ*H* is in (kJ/mol); Δ*S* is in (kJ/(mol·K)); *R* is the universal gas constant (8.314 J/mol); *T* is the adsorption temperature (K).

## 3. Results and Discussion

### 3.1. Characterizations

#### 3.1.1. Analysis of the Mechanism of Jute Fiber Modification

**Figure 1:**
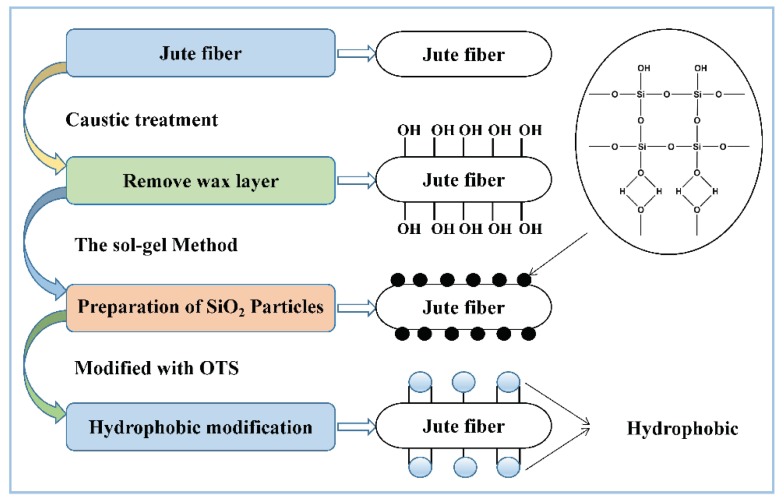
The fabrication of hydrophobic and lipophilic jute fibers.

The hydrophobic and lipophilic processes of jute fiber fabrication are described in Figure 1. In this study, caustic pretreatment is done to remove the layer of plant fiber wax, to expose more hydroxyls, and to improve the interface bonding properties. TEOS hydrolyzes as SiO_2_ particles and adheres to the surface of the material via a chemical effect. Finally, the material is immersed in OTS/n-hexane solution. Since the silica surface is coated with silane, the long-chain hydrophobic alkyls are introduced onto the jute fiber surface; this makes the fiber resistant to wetting by water droplets [8,23].

#### 3.1.2. FTIR Spectra and BET Analysis

**Figure 2:**
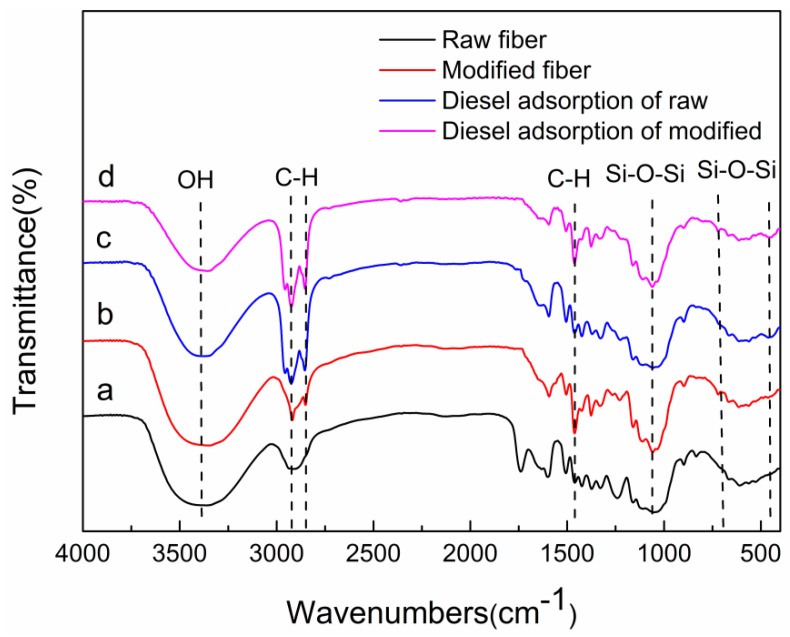
Fourier transform infrared (FTIR) spectra of (**a**) raw jute fiber; (**b**) octadecyltrichlorosilane (OTS) modified; (**c**) diesel adsorption of raw and (**d**) diesel adsorption of modified.

The FTIR spectra of raw and modified jute fibers are shown in Figure 2. Compared with the peak of the raw jute fiber, that of modified jute fiber at 470 cm^−1^ and the new absorption peaks at 798 and 1112 cm^−1^ are markedly strengthened; these correspond to Si–O–Si bending, indicating that SiO_2_ has adhered to the jute fiber surface [8,24,25]. The vibration of –C–H (–CH_2_ and –CH_3_) groups are observed at 1430, 2920, and 2855 cm^−1^. This indicates that OTS and jute have been successfully bonded [26,27]. The peaks at 3430 cm^−1^ are attributed to –OH stretching vibrations [8,28]. After diesel adsorption, there are no new groups generated, indicating that the adsorption of diesel onto fibers is not through chemical bonding, physical adsorption is dominant [29].

The total surface areas (Sp) of the jute fiber were calculated based on the BET model, the specific surface areas of fibers were measured and are shown in Table 1.

**Figure 3:**
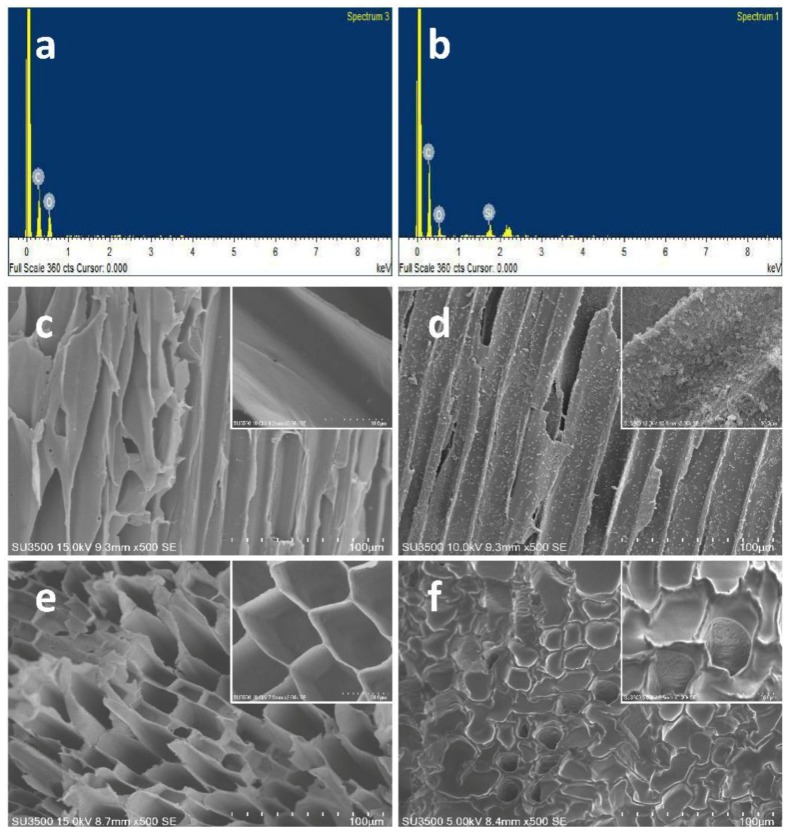
Energy-dispersive spectrometry (EDS) spectra of (**a**) raw and (**b**) modified fibers; scanning electron microscopy (SEM) images of raw (**c**) and modified (**d**) fibers; porous structure before (**e**) and after adsorption (**f**) of diesel oil.

The specific surface area of the modified material is much larger than the raw fiber, because the SiO_2_ particles attached to the surface contribute to the increase of its specific surface area, which is more conducive to aiding the oil adsorption capacity. After diesel adsorption, there is nearly no difference between the raw and modified fiber. For the modified fiber, the specific surface area after adsorption is much smaller than before, the large deviation in the calculated specific surface area may be caused by the oil plug in the porous structure. (Figure 3f) [29,30,31].

#### 3.1.3. EDS and SEM Analysis

The chemical compositions of raw and modified jute were characterized by EDS, as shown in Figure 3a,b. The EDS spectrum of raw jute shows the presence of the elements C and O (Figure 3a). In case of the fibers modified with SiO_2_ and OTS, the peak for Si can also be observed along with those for C and O (Figure 3b). This result reveals that raw fiber is modified successfully with SiO_2_ and OTS. In order to further study the morphological changes after modification, raw and modified fiber were analyzed by SEM (results are displayed in Figure 3c,d). The results prove that the surface of the raw fiber is relatively smooth (Figure 3c). The magnified image of the fiber also shows a smooth surface of the raw fiber. After coating with SiO_2_ particles and modification with OTS, the surface becomes much rougher than that of the raw fiber (Figure 3d). The magnified image displays more clearly the dispersion and morphology of SiO_2_ particles on the surface fiber at higher magnification. The jute fiber has a smooth structure, a thin fiber wall, and a clear porous structure (Figure 3e) before diesel adsorption. After adsorbing, a thick oil film is attached to the surface of the fiber, and oil is also attached to the internal fiber walls of the gap. As a result, the entire fiber wall becomes thicker and most of the porous structure is blocked (Figure 3f) [29].

#### 3.1.4. Wettability Analysis

**Figure 4:**
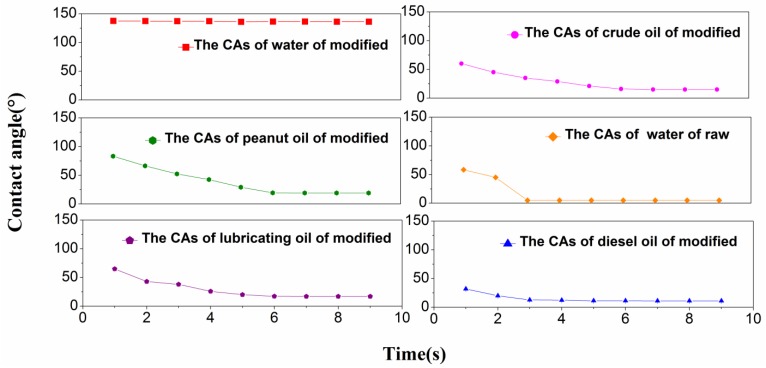
The contact angles (CAs) of different liquids.

Figure 4 shows that CAs of different liquids change on the fiber surfaces. All of the oil CAs after modification are less than 20° when they reach stable states. According to Young’s equation [32], the modified jute fiber has better wettability for different oils and water CAs of up to 136.2° indicate high hydrophobicity. Because the CA of the water drop on the surface of the raw fibers is too small to be determined by the CA goniometer, the wetted area has to be measured. Therefore, the effective CA is obtained on the basis of the spherical cap. The wetted area of a 3 μL drop is about 20 mm^2^; therefore its CA is about 5° [33].

### 3.2. Analysis of Oil Adsorption and Water Adsorption of Modified Jute Fiber in an Oil System and in a Water System

**Figure 5:**
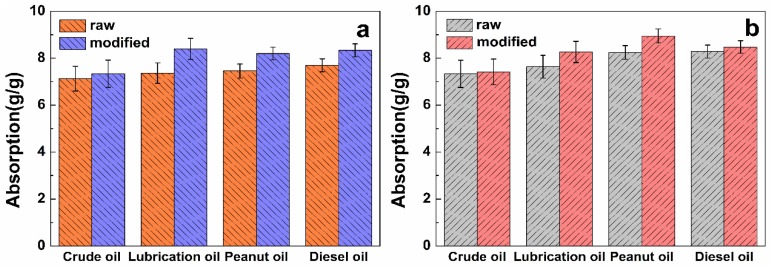
(**a**) Rapid adsorption and (**b**) saturated adsorption in a pure oil system.

As shown in Table 2, the adsorption capacity of raw and modified fibers approach the maximum value in 15 min, increasing slowly thereafter. After 40 min, the oil and water adsorbed in the fiber lumen reach maximum saturation. After 24 h, adsorption is almost unchanged as compared with that at 40 min. The rapid water adsorption of jute fiber decreases from 6.37 ± 0.06 g/g (raw) to 0.55 ± 0.04 g/g (modified), and the saturated water adsorption decreases from 8.53 ± 0.03 g/g to 0.92 ± 0.02 g/g, indicating excellent hydrophobicity. For the raw jute fiber, the rapid adsorption capacities for crude oil, lubricating oil, peanut oil, and diesel oil are 7.12 ± 0.05 g/g, 7.36 ± 0.04 g/g, 7.45 ± 0.06 g/g, and 7.50 ± 0.02 g/g, respectively. The adsorption rate of the modified fiber increases by 1.03, 1.14, 1.1, and 1.11 times, respectively (Figure 5a). On the other hand, the saturated adsorptions are 7.33 ± 0.07 g/g, 9.53 ± 0.05 g/g, 8.24 ± 0.04 g/g, and 8.38 ± 0.05 g/g, respectively. After modification, they are 7.41 ± 0.03 g/g, 10.29 ± 0.04 g/g, 8.94 ± 0.02 g/g, and 8.48 ± 0.04 g/g, respectively (Figure 5b). Hydrophobicity increases greatly after modification of the raw fiber, and lipophilicity has a certain degree of improvement. All representative results are in g/g (g (liquid)/g (sorbent)).

The maximum adsorption capacity of modified jute fiber was compared with the other adsorbents reported used for diesel oil adsorption in the literature (Table 3) [8,29,34,35,36,37,38,39,40]. It was found that the maximum adsorption capacity of the modified fiber is higher than most of the inorganic adsorbents. Although, some adsorbents had better adsorption capacity than modified jute, they are expensive and difficult to biodegrade. So, on the basis of cost and biodegradability, modified jute fiber has better prospects.

### 3.3. Adsorption Kinetic

**Figure 6:**
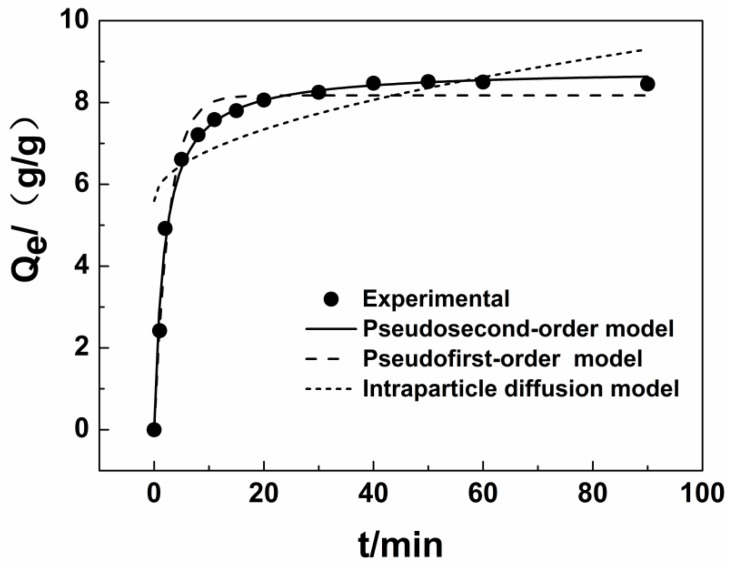
Kinetics of diesel adsorption on modified jute fiber.

An example of the adsorption kinetics curve for the modified jute (diesel adsorption) is shown in Figure 6. The adsorption rate reflects the amount of modified jute in the oil–water system per unit time. The slope at each point represents the instantaneous adsorption rate. The amount of adsorption increases rapidly (in 8 min). The amount adsorbed increases slowly from 8 to 40 min, and the adsorption equilibrium is reached after 40 min. The reason for this phenomenon is attributed to the large number of internal pores in the modified jute structure. Because diesel is a hydrophobic viscous material and the solubility in water is very low, the diesel can quickly adsorb onto the surface of the material through intermolecular forces. Meanwhile, the diesel is transported into the interior of the jute fiber because of capillary action. The diffusion then becomes more difficult as the pore volume within the fiber decreases, so the amount adsorbed tends to increase very slowly and finally reaches a steady state.

In this study, pseudo-first-order and pseudo-second-order adsorption kinetic models and intraparticle diffusion kinetics model were used to fit the data (Table 4). The correlation coefficient (R^2^) for the equation for intraparticle diffusion kinetics is less than 0.6, indicating that the adsorption process does not fit the kinetic model. The equilibrium adsorption capacities calculated by using the equations for pseudo-first-order and pseudo-second-order adsorption kinetics are 8.17 ± 0.01 g/g and 8.81 ± 0.01 g/g, respectively; the equilibrium adsorption capacity from measurement is 8.48 ± 0.02 g/g. The calculated equilibrium adsorption capacity is close to the experimental values, but the pseudo-second-order adsorption kinetics model has a higher R^2^ (0.9918). Therefore, the pseudo-second-order kinetic equation is more suitable for describing diesel adsorption onto jute fiber.

### 3.4. Adsorption Isotherm

**Figure 7:**
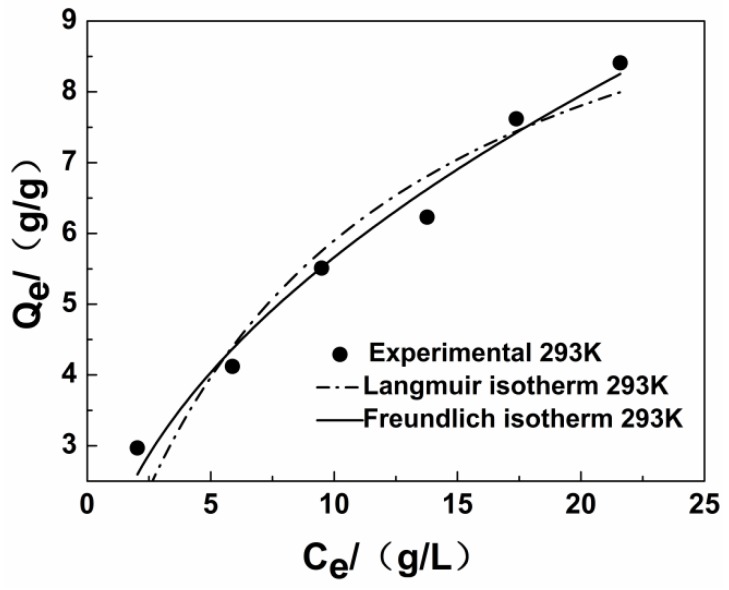
Adsorption isotherm of diesel onto modified fiber.

The mathematical model for the adsorption isotherm for modified jute fiber in an oil/water mixture at 293 K is presented in this part. The results are shown in Figure 7 and Table 5. Comparison of the R^2^ values (Table 5) reveals that the Freundlich model is the best fitting model to explain the adsorption of diesel from modified jute fibers. This result shows that the adsorption of diesel onto the modified fibers studied is multilayer adsorption, and the Freundlich index 1/n is greater than about 0.5, indicating that the material is a favorable absorbent [41].

### 3.5. Adsorption Thermodynamics

**Figure 8:**
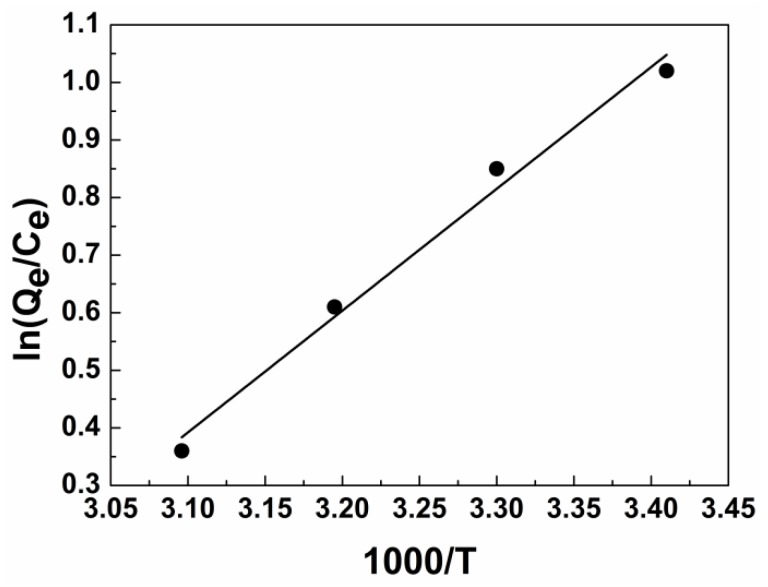
Plot of In(*Q_e_*/*C_e_*) versus 1000/T for diesel adsorption on modified fiber for thermodynamic parameters.

The values of the thermodynamic parameters Δ*G* and Δ*H* can be calculated by plotting ln(*Qe*/*Ce*) against 1000/T (Figure 8 and Table 6). Δ*G* values are between −2.48 and −0.09, indicate that in the adsorption process, diesel molecules tend to spontaneously adsorb from the oil/water mixture to the surface of modified material; the absolute value of Δ*G* decreases with increasing temperature. In other words, higher temperature and weaker driving force of adsorption lead to more difficult absorption of diesel. If Δ*S* < 0, then the movement of diesel molecules in the modified fiber is limited, the arrangement of molecules is more orderly, and ΔH decreases. On the other hand, Δ*H* < 0 indicates that diesel adsorption is essentially an exothermic process (|Δ*H*| < 41 kJ/mol). We consider that the adsorption of diesel oil onto jute occurs through van der Waals forces, the consequence is identical with the results of FTIR.

## 4. Conclusions

Imparting a rough structure to the surface of jute fiber through the sol–gel method is feasible and effective. Using a modified hydrophobic or oleophilic material, silane, is also practical and useful for marine oil spill cleanup. Compared with other modified materials, natural fiber has sufficient buoyancy and less pollution potential, it is degradable by microorganisms, and it is environment-friendly. For this paper, the capacity for adsorption of different oils and water onto modified jute fiber were studied. The adsorption capacity for water decreased from 8.53 ± 0.03 g/g to 0.92 ± 0.02 g/g after modification. The CA reached 136.2°, which greatly increases hydrophobicity. The adsorption of different oils also showed a certain degree of improvement. For example, the kinetics, isothermal equation, and thermodynamic parameters for diesel fuel adsorption onto modified fiber were analyzed. Kinetic data showed that the pseudo-second-order kinetic equation can better describe the adsorption process, and that gap diffusion affects the adsorption rate. Compared with the Langmuir adsorption isotherm, the Freundlich equation can better describe the equilibrium adsorption data, indicating multilayer adsorption of diesel oil onto the modified jute fiber. The thermodynamic parameters prove that the adsorption of diesel onto modified jute is a spontaneous and favorable process.

## Figures and Tables

**Table 1:** The specific surface area of the fiber before and after adsorption of diesel oil.

Fiber	BET Surface Area
Raw fiber	0.17 ± 0.06 m²/g
Modified fiber	63.84 ± 0.47 m²/g
Raw fiber after diesel adsorption	0.23 ± 0.01 m²/g
Modified fiber after diesel adsorption	0.48 ± 0.01 m²/g

**Table 2:** Water adsorption of raw and modified jute.

**State**	**5**	**10**	**15**	**20**	**25**	**30**
Raw	4.23 ± 0.02	5.44 ± 0.04	6.37 ± 0.06	7.12 ± 0.02	7.83 ± 0.04	8.51 ± 0.03
Modified	0.32 ± 0.01	0.43 ± 0.02	0.55 ± 0.02	0.61 ± 0.03	0.79 ± 0.02	0.81 ± 0.02
**State**	**35**	**40**	**45**	**50**	**55**	**60**
Raw	8.52 ± 0.04	8.52 ± 0.04	8.52 ± 0.03	8.53 ± 0.05	8.53 ± 0.02	8.53 ± 0.03
Modified	0.82 ± 0.01	0.83 ± 0.03	0.91 ± 0.01	0.91 ± 0.02	0.92 ± 0.01	0.92 ± 0.02

**Table 3:** Comparative adsorption capacities of various adsorbents for diesel adsorption.

Adsorbents	Maximum Diesel	References
	Adsorption	
	Capacity (g/g)	
Raw cotton fiber	15	[8]
Mesoporous silica aerogel	13.6	[29]
Surfactant grafted PDA-PAN nanofiber	62.53	[34]
The elastic cellulose-based aerogels	91.82	[35]
Sponge treated by trisilanophenyl POSS	8.9	[36]
Cotton modified using P-SiO_2_ nanoparticles	20	[37]
Kapok modified using P-SiO_2_ nanoparticles	23	[37]
Modified hygroscopic magnesium carbonate	3.017	[38]
Barium sulfate sorbent powder	1.6	[39]
Treated bark	2	[40]
Jute fiber modified via the sol-gel method	8.48	This study

**Table 4:** Kinetic parameters for diesel adsorption from an oil/water mixture using modified fiber.

Kinetic Model	Parameters	Value
Pseudofirst-order	*Q_e_*	8.1710
	*K*_1_	0.3700
	R^2^	0.9805
Pseudosecond-order	*Q_e_*	8.8137
	*K*_2_	0.0603
	R^2^	0.9918
Intraparticle diffusion	*K*_3_	0.3904
	*C*	5.5945
	R^2^	0.5449

**Table 5:** Thermodynamic parameters of diesel adsorption onto modified fiber.

Isotherm Model	Isotherm Constants	Temperature (293 K)
Langmuir	*Q*_0_	11.5209
	*K*_1_	0.1050
	R^2^	0.9075
Frenudlic	*n*	2.0437
	*K*_2_	1.8355
	R^2^	0.9753

**Table 6:** Thermodynamic parameters for diesel adsorption on modified jute fiber.

T/K	Ln (*Q_e_*/*C_e_*)	ΔG/kJ·mol^−1^	ΔH/kJ·mol^−1^	ΔS/J·mol^−1^·k^−1^	R^2^
293	1.02	−2.48	−17.54	−51.21	0.9821
303	0.85	−2.14			
313	0.61	−1.59			
323	0.36	−0.97			
